# Deprescribing and Anti-CGRP preventive therapy in medication-overuse headache associated with migraine: pathophysiological rationale and single-center real world evidence

**DOI:** 10.3389/fdsfr.2026.1836444

**Published:** 2026-08-12

**Authors:** Matija Sošić, Valentino Rački, Almir Fajkić, Mladen Jašić, Andrej Belančić

**Affiliations:** 1 Special Hospital for Orthopedics and Rehabilitation “Martin Horvat” Rovinj-Rovigno, Rovinj, Croatia; 2 Faculty of Medicine, Juraj Dobrila University of Pula, Pula, Croatia; 3 Department of Neurology, Clinical Hospital Centre Rijeka, Rijeka, Croatia; 4 Department of Neurology, Faculty of Medicine, University of Rijeka, Rijeka, Croatia; 5 Department of Pathophysiology, Faculty of Medicine, University of Sarajevo, Sarajevo, Bosnia and Herzegovina; 6 Department of Basic and Clinical Pharmacology and Toxicology, University of Rijeka, Faculty of Medicine, Rijeka, Croatia

**Keywords:** analgesics, calcitonin gene-related peptide, headache, medication overuse, migraine disorders, monoclonal antibodies, neuroplasticity

## Abstract

Medication-overuse headache (MOH) associated with migraine is a biologically driven disorder involving central sensitization, maladaptive neuroplasticity, and enhanced calcitonin gene-related peptide (CGRP) signaling. While structured deprescribing addresses medication-related drivers, withdrawal alone often provides limited and unsustained benefit. Anti-CGRP preventive therapies offer targeted modulation of migraine pathways without reinforcing medication overuse. This study integrates pathophysiological insights with real-world evidence from a single-center study of 47 patients treated with combined deprescribing and anti-CGRP therapy. Clinically meaningful reductions in monthly migraine days were observed, with 85.1% achieving ≥50% reduction at 6 months and 78.7% achieving >75% reduction at 12 months. Our clinical experience also suggests that long-standing combination analgesic overuse may be associated with a slower treatment response. The present findings support a mechanism-based treatment approach and suggest that combining preventive therapy with deprescribing may optimize long-term outcomes in patients with migraine-associated MOH.

## 1 Introduction

Medication-overuse headache (MOH) associated with migraine is a complex secondary headache disorder arising from sustained exposure to acute headache medications and the induction of maladaptive neuroplastic changes that promote headache chronification (Ashina et al., 2023). Rather than representing a purely behavioral consequence of excessive medicine intake, MOH is increasingly recognized as a biologically mediated condition in which repeated pharmacological exposure alters central nociceptive processing, impairs descending pain inhibition, and reinforces sensitization mechanisms. In patients with migraine, these processes interact with disease-specific pathophysiology, resulting in escalating headache frequency, reduced responsiveness to acute treatments, and substantial long-term disability (Ljubisavljevic et al., 2023).

From a clinical pharmacology perspective, MOH occupies a unique position at the intersection of therapeutic benefit and -induced pathology. Acute migraine medications are effective in aborting individual attacks, but if used repeatedly, can reinforce pronociceptive signaling and maladaptive neural circuits. A major paradigm shift in migraine prevention has been achieved with the introduction of monoclonal antibodies targeting calcitonin gene-related peptide (CGRP) or its receptor, including fremanezumab, galcanezumab, erenumab, and eptinezumab, along with gepants; atogepant, and rimegepant. Conversely, therapies targeting CGRP, offer targeted modulation of molecular pathways central to migraine pathophysiology without perpetuating medication overuse behavior (Pellesi et al., 2026). This duality underpins contemporary approaches integrating deprescribing strategies with mechanism-based preventive therapy.

This Perspective examines the pathophysiological and pharmacological underpinnings of MOH associated with migraine, emphasizing medication-induced sensitization and dysregulated CGRP signaling. Within this framework, we discuss the rationale for structured deprescribing, the role of anti-CGRP preventive therapy, and contextualize these concepts using real-world observational evidence from a single tertiary headache center.

## 2 Pathophysiological basis of medication-overuse headache

MOH is characterized by altered nociceptive processing that extends beyond peripheral trigeminovascular activation. Experimental and clinical evidence indicates that repeated exposure to acute headache medications lowers nociceptive thresholds, facilitates central sensitization, and weakens descending inhibitory pain control. Mechanistically, dysfunction of descending control pathways linking the periaqueductal gray and rostroventromedial medulla, along with monoaminergic imbalance (serotonergic and noradrenergic) that can shift the system toward facilitation rather than inhibition (Bannister and Dickenson, 2017). Beyond neuronal mechanisms, glial activation (microglia and astrocytes) and low-grade neuroinflammatory signaling have been implicated in maintaining a sensitized state, contributing to the persistence of pain facilitation even after the acute trigger is removed (Inoue and Tsuda, 2018). Functional and structural neuroimaging studies report altered metabolism and connectivity in cortical and subcortical regions involved in pain modulation, affective processing, and reward-related behavior, including the orbitofrontal cortex, anterior cingulate cortex, amygdala, thalamus, and mesolimbic dopaminergic circuitry, consistent with the involvement of medication-related reinforcement mechanisms (Pellesi et al., 2026).

At the neurochemical level, class-dependent changes have been described with chronic intake of analgesics, triptans, opioids, or combination medications, and have been linked to altered serotonergic transmission, changes in endogenous opioid signaling, and enhanced pronociceptive activity. Experimental models demonstrate that sustained exposure to acute migraine medications increases cortical excitability and susceptibility to cortical spreading depolarization while upregulating CGRP and other neuropeptides within the trigeminovascular system (Ljubisavljevic et al., 2023), supporting a role for CGRP-pathway amplification in chronification. These changes provide a mechanistic link between medication overuse and migraine chronification. Importantly, MOH-related neuroplasticity appears partly reversible. Clinical and imaging studies indicate partial normalization of abnormal metabolic activity following the successful withdrawal of overused medications, supporting the concept of MOH as a pharmacologically modifiable disorder (Wang et al., 2025). However, reversibility is often incomplete in patients with long-standing chronic migraine, underscoring the need for adjunctive preventive strategies that target persistent sensitization biology, particularly CGRP-mediated trigeminovascular signaling.

## 3 Pharmacological rationale for deprescribing in MOH

Deprescribing is defined as structured withdrawal or restriction of overused acute medications and remains a cornerstone of MOH management. From a pharmacodynamic standpoint, deprescribing aims to remove exogenous drivers of pronociceptive plasticity and restore endogenous pain modulation. Clinically, this approach reduces exposure to medications that perpetuate sensitization and mitigates the risk of medication-related adverse effects.

Despite its central role, deprescribing alone frequently yields suboptimal outcomes. Randomized controlled trials and meta-analyses demonstrate that abrupt withdrawal without concomitant preventive therapy results in modest and inconsistent reductions in monthly headache days and is associated with poor tolerability and high relapse rates (Pellesi et al., 2026). These findings reflect the multifactorial nature of MOH, in which medication overuse acts as a perpetuating factor but does not fully account for entrenched migraine pathophysiology.

Consequently, contemporary treatment paradigms increasingly emphasize integrated strategies combining deprescribing with early initiation of preventive therapy, aiming to stabilize the underlying biological drivers of migraine while removing headache-promoting pharmacological exposure (Pensato et al., 2022). On the other hand a real world experience study in 200 patients, does not support the hypothesis that a CGRP-mAbs plus detoxification strategy should be the therapeutic benchmark in severe CM + MOH. Instead, the findings suggest a paradigm shift in which CGRP-mAbs alone may be effective, making mandatory withdrawal of overused medications less essential. As migraine frequency decreases under preventive therapy, acute medication use may progressively decline without abrupt discontinuation and its associated withdrawal symptoms. Given the clinical and socio-economic burden of CM + MOH and the demonstrated efficacy of CGRP-mAbs, their use as a first-line strategy should be considered, alongside a reconsideration of routine deprescribing and the stigma implied by the term “detoxification” (Silvestro et al., 2025).

## 4 Anti-CGRP preventive therapy as mechanism-based treatment in MOH

Monoclonal antibodies targeting CGRP or its receptor represent a major advance in migraine prevention with particular relevance for MOH. CGRP is a key mediator of trigeminovascular activation, peripheral and central sensitization, and neurogenic inflammation—processes that are amplified by repeated acute medication exposure. Anti-CGRP therapies exhibit several pharmacological properties that distinguish them from traditional oral preventives. Their high target specificity, minimal interaction with hepatic medication-metabolizing enzymes, and stable pharmacokinetics allow predictable exposure with infrequent dosing. Importantly, they do not engage central reward pathways or reinforce medication-seeking behavior, making them particularly suitable for patients with medication overuse (Wattiez et al., 2020).

Systematic reviews and meta-analyses demonstrate that anti-CGRP monoclonal antibodies significantly reduce monthly migraine days, improve ≥50% response rates, and facilitate reversion from MOH to non-MOH status in patients with chronic migraine. Compared with traditional preventive agents, effect sizes are generally larger and associated with lower discontinuation rates. Network meta-analytic evidence further indicates that combination strategies, incorporating preventive therapy alongside medication restriction or withdrawal, are superior to withdrawal alone in reducing headache burden and preventing relapse (Koonalintip et al., 2025).

These data support the use of anti-CGRP therapy not only as migraine prevention but also as a stabilizing pharmacological intervention during deprescribing, mitigating withdrawal-related exacerbations and reducing the likelihood of relapse.

## 5 Clinical landscape and barriers to first-line use of Anti-CGRP therapies

Although anti-CGRP monoclonal antibodies are supported by high-quality evidence and strong guideline recommendations for migraine prevention (Table 1), their positioning as first-line therapy remains inconsistent across healthcare systems. Recent evidence-based guidelines for the pharmacological treatment of migraine assign strong recommendations to multiple anti-CGRP agents in both episodic and chronic migraine, reflecting robust efficacy and favorable tolerability profiles (Ornello et al., 2025).

**TABLE 1:** Approved medications and pivotal trials acting on the CGRP system.

Brand name	INN (molecule)	Drug class	Target	Route of administration	Regulatory status in Europe	Pivotal phase III trials
Aimovig	Erenumab	Monoclonal antibody	CGRP receptor	Subcutaneous (monthly)	EMA approved; widely used in EU	Strive; arise; liberty
Emgality	Galcanezumab	Monoclonal antibody	CGRP ligand	Subcutaneous (monthly)	EMA approved	EVOLVE-1; EVOLVE-2; regain
Ajovy	Fremanezumab	Monoclonal antibody	CGRP ligand	Subcutaneous (monthly/quarterly)	EMA approved	HALO EM; HALO CM; focus
Vyepti	Eptinezumab	Monoclonal antibody	CGRP ligand	Intravenous (quarterly)	EMA approved	promise-1; promise-2
Vydura/Nurtec ODT	Rimegepant	Gepant	CGRP receptor	Oral	EMA approved	Study 301; study 302; BHV3000-305
Aquipta/Qulipta	Atogepant	Gepant	CGRP receptor	Oral daily	Used in several EU countries (national access varies)	Advance; progress

Despite this, reimbursement policies in many countries continue to require failure of several conventional oral preventives before access to anti-CGRP therapy is granted. Such step-therapy requirements are largely driven by economic and administrative considerations rather than pathophysiological rationale. Notably, only a limited number of European countries currently provide reimbursement for anti-CGRP monoclonal antibodies as a first-line preventive option, based primarily on disease burden and clinical judgment rather than sequential treatment failure. These include Croatia, Iceland, Lithuania, and Luxembourg (Jančuljak et al., 2024; Remenčiūtė, 2024; ISPOR Europe, 2025; Luxembourg, 2026).

From a pharmacological and pathophysiological perspective, such reimbursement models are well aligned with the current understanding of migraine and medication-overuse headache (MOH). Early access to anti-CGRP preventive therapy facilitates rapid stabilization of CGRP-mediated sensitization, reduces reliance on acute medications, and supports timely and effective deprescribing strategies. In patients with established or incipient MOH, this approach enables earlier interruption of medication overuse cycles, potentially shortening disease duration, reducing relapse risk, and limiting further chronification.

In contrast, delayed access to anti-CGRP therapies in systems with restrictive reimbursement policies may inadvertently prolong exposure to overused acute medications, sustain central sensitization, and undermine the effectiveness of deprescribing efforts. These disparities highlight the need for greater alignment between reimbursement frameworks, guideline-level evidence, and migraine pathophysiology across Europe.

## 6 Pediatric and adolescent pharmacological considerations

Migraine and medication-overuse headache are increasingly recognized in pediatric and adolescent populations, where frequent use of acute medications such as NSAIDs and triptans represent a major risk factor for headache chronification. Migraine has a prevalence is up to 20% in children and adolescents (Abu-Arafeh et al., 2010). In chronic migraine, the effectiveness of acute treatments tends to be reduced. Coexisting factors such as obesity and neuropsychiatric comorbidities may contribute to diminished responsiveness to acute medications (Saracco et al., 2014). Evidence from pediatric research indicates that medication overuse headache (MOH) occurs less frequently in children and adolescents compared to adult populations (Moavero et al., 2020; Papetti et al., 2019; Langdon and DiSabella, 2017; Stevens et al., 2014; Heyer and Idris, 2014). A recent study suggests that the reduced response to analgesics in patients with chronic migraine does not account for the relatively low prevalence of MOH. It also reports that preventive therapy may improve the effectiveness of acute medications and lessen migraine-related disability, while psychiatric comorbidities, although relevant for overall assessment, do not appear to influence acute treatment response (Frattale et al., 2024).

Evidence-based guidelines emphasize limitation of acute medication intake in younger patients, given the well-established association between frequent analgesic use and development of MOH. Preventive pharmacological options in pediatric migraine have historically been limited by inconsistent efficacy and tolerability of traditional agents. While topiramate and other antiseizure medications have been used, evidence supporting their efficacy in migraine with medication overuse remains limited and inconsistent. Similarly, although botulinum toxin type A is strongly recommended for chronic migraine in adults, pediatric data remain sparse and largely extrapolated. Recent regulatory approval of fremanezumab for the preventive treatment of episodic migraine in pediatric patients represents a significant pharmacological milestone (Ornello et al., 2025). Given the central role of CGRP in migraine pathophysiology and sensitization processes underlying MOH, mechanism-based preventive therapies targeting the CGRP pathway may hold particular promise for reducing both migraine frequency and the risk of medication overuse in younger populations. Although formal studies specifically addressing MOH in pediatric patients treated with anti-CGRP agents are currently lacking, biological plausibility and favorable safety profiles support further investigation in this vulnerable group.

## 7 Real-world evidence based on a single-centre experience

To complement mechanistic and trial-derived evidence, real-world data provide critical insight into the effectiveness and feasibility of integrated deprescribing and anti-CGRP preventive therapy in routine clinical practice.

### 7.1 Single-center observational data

To support the review, we included a descriptive single-center observational series from our tertiary Headache Clinic. Adult patients with chronic migraine and medication-overuse headache were treated with a structured deprescribing approach based on national expert recommendations (Hucika and Petravić, 2020). This approach included stopping or limiting the overused acute medications, with the specific strategy individualized according to the patient’s clinical presentation and the treating physician’s judgment, alongside initiation of anti-CGRP preventive therapy when clinically appropriate. Follow-up was performed during routine clinical visits, and the main outcomes were monthly headache days, acute medication use, and MOH status over time. The purpose of this dataset was to show how this strategy works in real clinical practice, rather than to provide evidence from a formal hypothesis-testing cohort study.

Forty-seven of 50 patients (36 women and 11 men) completed follow-up and were included in the analysis, while three were excluded because they were lost to follow-up. Forty-nine percent of patients overused an over-the-counter combination analgesic containing paracetamol, propyphenazone, caffeine, and codeine, 32% overused triptans, and 19% overused paracetamol, ibuprofen, or a tramadol-paracetamol combination. Anti-CGRP treatment was selected according to routine clinical practice. Because anti-CGRP medicatinos are generally considered to have similar efficacy as a class, and because the subgroups were small, we did not perform a separate analysis for each individual medication.

### 7.2 Statistics

Collected data were extracted to Microsoft Excel 2016 (Microsoft Office) and MedCalc v12.1.3 (MedCalc Software bvba, Ostend, Belgium), which were subsequently used for statistical analysis. Kolmogorov-Smirnov test was used to assess the normality of distribution. Variable values were described by absolute and relative frequencies, measures of central tendency and measures of spread. Changes over time (baseline vs. at 6 months vs. at 12 months) in terms of number of monthly headache days, after anti-CGRP medicine introduction plus deprescribing measures, were analysed with Friedman test. Reduction in the average monthly headache days after 6 and 12 months was also presented from the perspective of percentage improvement from baseline (<50%, 50%–74%, and >75% reduction in monthly headache days).

### 7.3 Results

Changes in monthly headache days were evaluated at 6 and 12 months after initiation of anti-CGRP therapy combined with deprescribing measures, expressed as the percentage reduction from baseline. We noticed a reduction in monthly migraine days at 6 and 12 months from the baseline visit (Supplementary Figure S1).

At 6 months, 14.9% (7/47) of patients achieved <50% reduction, 66.0% (31/47) achieved a 50%–74% reduction, and 19.1% (9/47) achieved >75% reduction in monthly headache days. At 12 months, only 2.1% (1/47) of patients remained in the <50% reduction category, 19.2% (9/47) achieved a 50%–74% reduction, and 78.7% (37/47) achieved >75% reduction.

At 12 months, response rates shifted toward greater improvement, with fewer patients achieving <50% or 50%–74% reduction and more patients achieving >75% reduction in monthly headache days (Table 2).

**TABLE 2:** Reduction in the average monthly headache days in the 6th and 12th months of treatment in patients with chronic migraine and medication overuse headache.

Percentage improvement	At 6 months		At 12 months	
	N	%	N	%
<50%	7	14.9%	1	2.1%
50%–74%	31	66.0%	9	19.2%
>75%	9	19.1%	37	78.7%
**Sum overall**	**47**	**100.0%**	**47**	**100.0%**

Preliminary observations indicate that this combined approach resulted in clinically meaningful reductions in headache frequency and medication overuse, with outcomes comparable to those reported in randomized controlled trials and other real-world cohorts. We also observed that patients with long-term overuse of combination analgesics tended to exhibit a slower favorable response to treatment. These findings support the feasibility and effectiveness of a mechanism-based strategy in everyday clinical settings.

## 8 Discussion

This perspective integrates pathophysiological insight, pharmacological rationale, and emerging clinical evidence to propose a mechanism-based approach to the management of medication-overuse headache (MOH) associated with migraine. Accumulating data indicates that MOH is not merely a behavioral phenomenon but a biologically mediated disorder driven by sustained pharmacological exposure and maladaptive neuroplasticity. Deprescribing remains essential to remove headache-promoting triggers; however, withdrawal alone often fails to address persistent sensitization and the underlying migraine biology.

In our patients, the combination of structured deprescribing with anti-CGRP preventive therapy resulted in a progressive and substantial reduction in monthly migraine days over time. At 6 months, most patients achieved a clinically meaningful response, with 66.0% reaching a 50%–74% reduction in monthly headache days, while 19.1% achieved a >75% reduction. Importantly, continued treatment was associated with further improvement, such that by 12 months nearly four-fifths of patients (78.7%) achieved a >75% reduction in monthly headache days. These findings suggest that the integration of mechanism-based preventive therapy with deprescribing may not only facilitate early improvement but also promote sustained clinical benefit over longer follow-up periods.

Our results are broadly consistent with growing evidence demonstrating the effectiveness of anti-CGRP monoclonal antibodies in patients with chronic migraine complicated by medication overuse. Reviews of CGRP-targeting therapies report robust reductions in monthly migraine days and high responder rates across clinical trials and real-world settings, accompanied by significant reductions in acute medication consumption (Makita et al., 2026). The mechanism underlying these benefits likely reflects direct modulation of trigeminovascular signaling and reduction of central sensitization, processes that are also implicated in the development and persistence of MOH.

Evidence from real-world observational studies further supports the clinical effectiveness of CGRP-targeted therapies in this population. In a large observational cohort of patients with chronic migraine and MOH, improvements in migraine frequency following initiation of anti-CGRP monoclonal antibodies were accompanied by a spontaneous decline in acute medication use (Tanei et al., 2024). Like our findings, these studies highlight the capacity of CGRP-targeted therapy to reduce headache burden and indirectly facilitate normalization of medication intake. However, while some real-world cohorts have reported improvements even without mandatory withdrawal, our data suggest that combining preventive therapy with structured deprescribing may lead to particularly robust long-term outcomes.

Furthermore, recent real-world data have questioned whether detoxification strategies are necessary when initiating anti-CGRP therapy. Silvestro et al. evaluated a cohort of 200 patients with chronic migraine and MOH treated with anti-CGRP monoclonal antibodies and found that structured detoxification did not provide additional clinical benefit compared with preventive therapy alone (Silvestro et al., 2025). These findings challenge the traditional paradigm in which withdrawal is considered an obligatory prerequisite for preventive treatment. While our study did not directly compare detoxification versus non-detoxification approaches, the substantial and progressive improvement observed in our patients suggests that combining deprescribing with targeted preventive therapy may remain a valuable strategy in selected patients, particularly in structured clinical settings.

Evidence from meta-analyses also support the effectiveness of CGRP-targeted therapy in patients with chronic migraine and medication overuse. A recent meta-analysis evaluating anti-CGRP monoclonal antibodies in this population demonstrated consistent reductions in monthly migraine days and acute medication used across multiple studies and treatment settings (Makita et al., 2026). Importantly, responder rates reported in several of the included studies are comparable to those observed in our patients, reinforcing the reproducibility of these therapeutic effects in real-world populations. Our findings extend this evidence by demonstrating that clinical improvement may continue to evolve over a 12-month period, suggesting that sustained blockade of CGRP signaling may progressively reverse sensitization mechanisms associated with migraine chronification and medication overuse.

From a pathophysiological perspective, these observations are biologically plausible. CGRP is an important mediator of trigeminovascular activation and plays a critical role in both peripheral and central sensitization processes. Chronic exposure to acute migraine medications may amplify these pathways, contributing to the persistence of headache and reinforcing medication overuse. By directly targeting CGRP signaling, monoclonal antibodies may attenuate trigeminal hyperexcitability, reduce neurogenic inflammation, and restore more physiological nociceptive processing. This mechanism-based approach addresses the underlying neurobiology of migraine chronification and may therefore be particularly effective in patients with MOH.

Taken together, our findings support a treatment paradigm in which mechanism-based preventive therapy is integrated with deprescribing strategies to address both the biological and behavioral drivers of medication overuse. While emerging evidence suggests that anti-CGRP therapy alone may be effective in many patients, the substantial and progressive improvements observed in our, indicate that combined approaches may offer additional clinical benefit, particularly in carefully managed clinical settings. Nevertheless, several questions remain unresolved. Most available evidence derives from observational cohorts, and randomized studies directly comparing detoxification strategies with immediate initiation of CGRP-targeted therapy are limited. Future prospective studies should aim to clarify optimal treatment sequencing, identify patient subgroups most likely to benefit from combined approaches, and determine whether early introduction of mechanism-based preventive therapy can reduce relapse rates and long-term disease burden. To the best of our knowledge, based on a targeted PubMed search, this represents one of the few real-world studies addressing this topic. Although the single-centre design may be considered a limitation, it is understandable due to the novelty of the report. Moreover, external validity is likely adequate, as patient management followed established guideline-based care and the study population was representative of typical patients with the investigated condition.

## 9 Conclusion

MOH associated with migraine reflects sustained pharmacological exposure leading to maladaptive neuroplasticity and CGRP-mediated sensitization. Although deprescribing addresses medication overuse, it is often insufficient as a standalone strategy. Anti-CGRP preventive therapies provide mechanism-based modulation of migraine pathways and may enhance outcomes either in combination with deprescribing or as a primary approach. While evolving evidence and recent regulatory shifts support reconsideration of treatment algorithms and earlier use of anti-CGRP therapies in patients at high risk of MOH, further adequately powered studies are needed to clarify whether monotherapy or combined strategies offer the greatest clinical benefit.

## Data Availability

The original contributions presented in the study are included in the article/Supplementary Material, further inquiries can be directed to the corresponding author.
