# Supplementary material for: Deprescribing and Anti-CGRP preventive therapy in medication-overuse headache associated with migraine: pathophysiological rationale and single-center real world evidence
Source: Front Drug Saf Regul. 2026 Aug 12;6:1836444. doi: 10.3389/fdsfr.2026.1836444 (PMC13506900; doi:10.3389/fdsfr.2026.1836444)
Supplement: Supplementary file 1 [file Supplementaryfile1.docx]

**Supplementary figure 1.** Changes over time (baseline vs at 6 months vs at 12 months) in terms of number of monthly headache days, after anti-CGRP medicine introduction plus deprescribing measures
